# Correction: Park et al. Real-World Outcomes of First-Line Chemotherapy in Metastatic Pancreatic Cancer: A Nationwide Population-Based Study in Korea. *Cancers* 2024, *16*, 3173

**DOI:** 10.3390/cancers17060963

**Published:** 2025-03-13

**Authors:** Chan Su Park, Byung Kyu Park, Joung-Ho Han, Kyong Joo Lee, Kang Ju Son

**Affiliations:** 1 Division of Gastroenterology, Department of Internal Medicine, National Health Insurance Service Ilsan Hospital, Goyang 10444, Republic of Korea; wpakcs@nhimc.or.kr; 2 Department of Internal Medicine, Chungbuk National University College of Medicine, Cheongju 28644, Republic of Korea; joungho@cbnu.ac.kr; 3 Division of Gastroenterology, Department of Internal Medicine, Hallym University Dongtan Sacred Heart Hospital, Hallym University College of Medicine, Hwaseong 18450, Republic of Korea; kyongjoolee1214@gmail.com; 4 Department of Policy Research Affairs, National Health Insurance Service Ilsan Hospital, Goyang 10444, Republic of Korea; sonkangju@nhimc.or.kr

## Error in Table

In the original publication [1], there was a mistake in Table 4 as published. The numerical values of the results described in the text differed from those presented in Table 4. The corrected Table 4 appears below. The authors apologize for any inconvenience caused and state that the scientific conclusions are unaffected. This correction was approved by the Academic Editor. The original publication has also been updated.

## Figures and Tables

**Table 4:** Hazard ratio of developing safety outcome events.

Outcomes	HR of Developing Events (95% CI)		
	GnP (*n* = 1134)	FOLFIRINOX (*n* = 1134)	*p*-Value
Emergency center visit	1.0 (ref.)	1.046 (0.937–1.168)	0.4221
Febrile neutropenia	1.0 (ref.)	2.285 (1.864–2.802)	<0.0001
Hospitalization *	1.0 (ref.)	1.160 (1.056–1.274)	0.0019

* Excluding hospitalization for chemotherapy. Abbreviations: HR, hazard ratio; CI, confidence interval; GnP, gemcitabine plus nab-paclitaxel; ref., reference.
