# Supplementary material for: Real-World Outcomes of First-Line Chemotherapy in Metastatic Pancreatic Cancer: A Nationwide Population-Based Study in Korea
Source: Cancers (Basel). 2024 Sep 16;16(18):3173. doi: 10.3390/cancers16183173 (PMC11430066; doi:10.3390/cancers16183173)
Supplement: Supplementary file 1 [file cancers-16-03173-s001.zip › cancers-3130437-supplementary.pdf]

Supplementary Materials

Supplementary Tables

Table S1. Chemotherapy rate in patients with metastatic pancreatic cancer by age group

Age group (years)		2012	2013	2014	2015	2016	2017	2018	2019	Total
21–50	Total No.	156	155	197	163	165	163	179	168	1,346
	No. of CTx	106	102	110	101	107	113	134	120	893
	(%)	(67.9)	(65.8)	(55.8)	(62.0)	(64.8)	(69.3)	(74.9)	(71.4)	(66.3)
51–60	Total No.	379	358	426	487	476	507	545	558	3,736
	No. of CTx	245	216	254	291	299	354	352	390	2,401
	(%)	(64.6)	(60.3)	(59.6)	(59.8)	(62.8)	(69.8)	(64.6)	(69.9)	(64.3)
61–70	Total No.	607	579	612	660	743	769	873	883	5,726
	No. of CTx	289	293	337	328	412	468	523	545	3,195
	(%)	(47.6)	(50.6)	(55.1)	(49.7)	(55.5)	(60.9)	(59.9)	(61.7)	(55.8)
71–80	Total No.	698	667	815	801	915	944	990	973	6,803
	No. of CTx	192	162	202	196	287	277	355	396	2,067
	(%)	(27.5)	(24.3)	(24.8)	(24.5)	(31.4)	(29.3)	(35.9)	(40.7)	(30.4)
≥ 81	Total No.	226	270	277	329	388	426	542	549	3,007
	No. of CTx	11	7	12	20	14	31	35	35	165
	(%)	(4.9)	(2.6)	(4.3)	(6.1)	(3.6)	(7.3)	(6.5)	(6.4)	(5.5)
Total	Total No.	2,066	2,029	2,327	2,440	2,687	2,809	3,129	3,131	20,618
	No. of CTx	843	780	915	936	1,119	1,243	1,399	1,486	8,721
	(%)	(40.8)	(38.4)	(39.3)	(38.4)	(41.6)	(44.3)	(44.7)	(47.5)	(42.3)

Abbreviation: CTx, Chemotherapy

Supplementary Figures

Figure S1

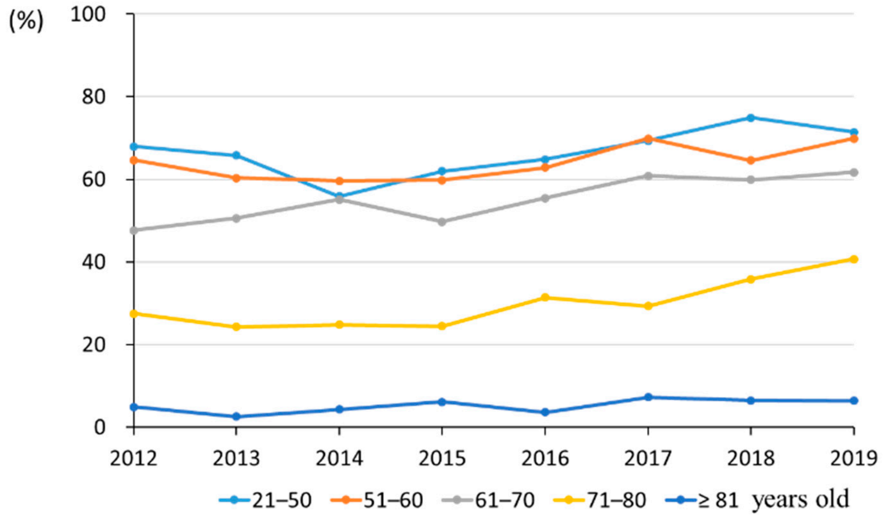

Figure S1. Annual rates of chemotherapy in patients with metastatic pancreatic cancer by age group.

Figure S2

A

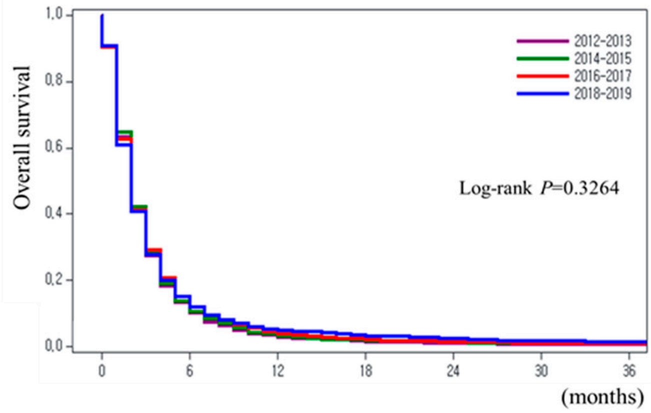

	Median OS (mo)	1 year (%)	2 years (%)	3 years (%)
2012–2013	2	3.4	1.2	0.7
2014–2015	2	3.8	1.5	1.0
2016–2017	2	4.7	1.6	1.0
2018–2019	2	5.5	2.5	1.3

B

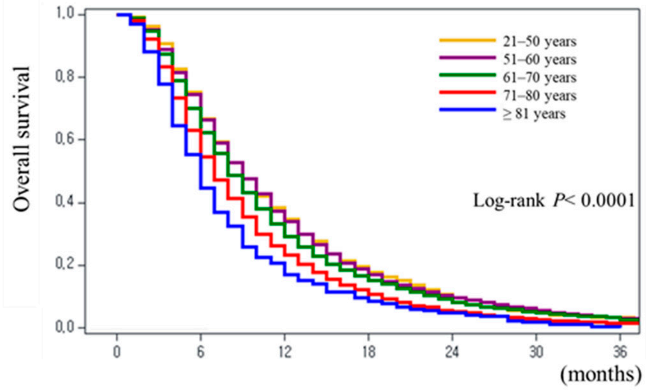

Age(years)	Median OS (mo)	1 year (%)	2 years (%)	3 years (%)
21–50	9	38.3	10.7	3.4
51–60	9	37.4	10.5	3.3
61–70	8	33.4	9.2	3.2
71–80	7	26.3	5.7	5.7
≥ 81	6	20.9	4.9	0.6

Figure S2. Overall survival of patients with metastatic pancreatic cancer. (A) Patients who did not receive chemotherapy, (B) Patients who received chemotherapy by age group.

Figure S3

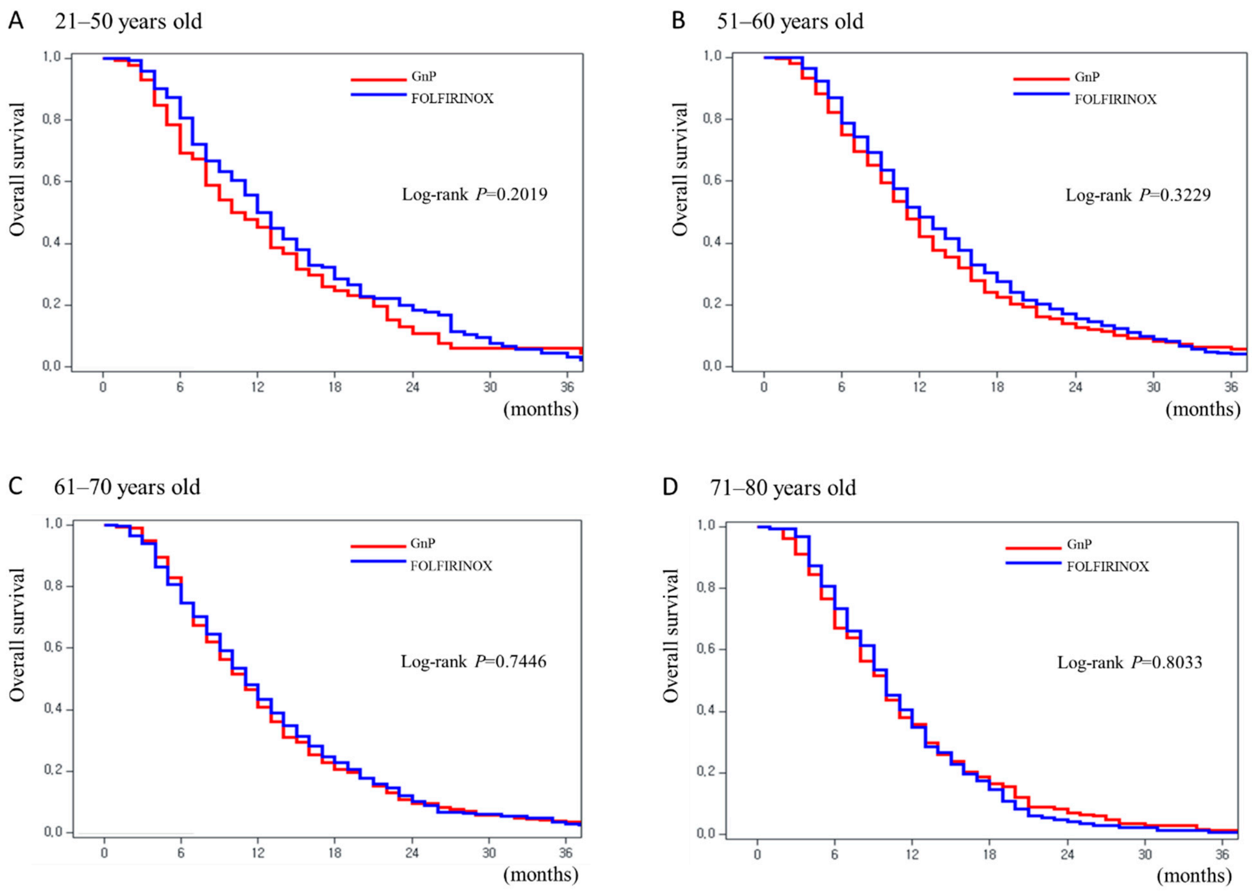

Figure S3. Overall survival of patients with metastatic pancreatic cancer receiving chemotherapy with gemcitabine plus nab-paclitaxel or FOLFIRINOX after propensity score matching by age group. (A) 21–50 years old, (B) 51–60 years old, (C) 61–70 years old, (D) 71–80 years old.
